# Air Change Rates and Interzonal Flows in Residences, and the Need for Multi-Zone Models for Exposure and Health Analyses

**DOI:** 10.3390/ijerph9124639

**Published:** 2012-12-12

**Authors:** Liuliu Du, Stuart Batterman, Christopher Godwin, Jo-Yu Chin, Edith Parker, Michael Breen, Wilma Brakefield, Thomas Robins, Toby Lewis

**Affiliations:** 1 School of Public Health, University of Michigan, Ann Arbor, MI 48108, USA; E-Mails: liuliudu@umich.edu (L.D.); ccgodwin@umich.edu (C.G.); jychin@umich.edu (J.-Y.C.); trobins@umich.edu (T.R.); 2 College of Public Health, University of Iowa, Iowa City, IA 51503, USA; E-Mail: edith-parker@uiowa.edu; 3 National Exposure Research Laboratory, Office of Research and Development, US Environmental Protection Agency, Research Triangle Park, NC 27711, USA; E-Mail: breen.michael@epa.gov; 4 Community Action Against Asthma, Community Partner at Large, Detroit, MI 48108, USA; E-Mail: ilovedawn14@sbcglobal.net; 5 School of Medicine, University of Michigan, Ann Arbor, MI 48108, USA; E-Mail: tobyl@med.umich.edu

**Keywords:** air change rate, air filters, interzonal flows, PM exposure, residences

## Abstract

Air change rates (ACRs) and interzonal flows are key determinants of indoor air quality (IAQ) and building energy use. This paper characterizes ACRs and interzonal flows in 126 houses, and evaluates effects of these parameters on IAQ. ACRs measured using weeklong tracer measurements in several seasons averaged 0.73 ± 0.76 h^−1^ (median = 0.57 h^−1^, n = 263) in the general living area, and much higher, 1.66 ± 1.50 h^−1^ (median = 1.23 h^−1^, n = 253) in bedrooms. Living area ACRs were highest in winter and lowest in spring; bedroom ACRs were highest in summer and lowest in spring. Bedrooms received an average of 55 ± 18% of air from elsewhere in the house; the living area received only 26 ± 20% from the bedroom. Interzonal flows did not depend on season, indoor smoking or the presence of air conditioners. A two-zone IAQ model calibrated for the field study showed large differences in pollutant levels between the living area and bedroom, and the key parameters affecting IAQ were emission rates, emission source locations, air filter use, ACRs, interzonal flows, outdoor concentrations, and PM penetration factors. The single-zone models that are commonly used for residences have substantial limitations and may inadequately represent pollutant concentrations and exposures in bedrooms and potentially other environments other where people spend a substantial fraction of time.

## 1. Introduction

With few exceptions, previous indoor air quality (IAQ) studies have assumed that residences and other small buildings are well mixed spaces throughout which pollutant concentrations are uniform, thus allowing the building to be represented as a single zone [1,2,3,4]. While this assumption facilitates analyses and may be reasonable for some purposes, it is highly idealized. The presence of spatial variation in pollutant concentrations, humidity levels and temperatures, and the mere presence of doors and walls separating building areas, suggest the limitations of this assumption. Even when building air appears reasonably well mixed, several factors, singly or jointly, may produce variation in pollutant levels that is important for exposure and other purposes like humidity control. First, the existence of localized emission sources within buildings, such as cigarette smoking or chemical use occurring in only portions of a building, is likely to produce pollutant differentials [4,5]. Second, pollutants such as particulate matter (PM) and ozone, which have relatively high deposition or removal rates, are sensitive to age-of-air and other factors that affect pollutant removal and lifetime [6,7] and that serve to increase spatial variation. Third, areas of buildings that are either partially decoupled from the rest of the building, or that have air change rates (ACRs) that differ from the building as a whole, may also differ in concentration, a result of varying rates of dilution, deposition or transport in the building [2,5]. These factors are especially relevant for pollutants that have localized sources and relatively high deposition rates, e.g., PM from cigarette smoking, and for persons spending considerable time in rooms that are decoupled from the general space, e.g., children or the elderly in bedrooms. 

The ACR is the volumetric flow of air into a room or building divided by the room or building volume, accounting for both airflow across the building envelope due to leakage and natural ventilation, as well as mechanical exchange, if any. The ACR represents a key variable for understanding IAQ, building energy consumption, and ventilation. Air change is the primary mechanism for entry of outdoor-generated air pollutants and for removal of indoor-generated air pollutants, and a minimum ACR is needed to dilute and remove pollutants emitted from indoor sources, e.g., PM emissions from smoking, cooking and vacuuming. In residences, determinants of ACRs include occupant behavior, e.g., window or door opening [8,9]; characteristics of the heating, ventilation and air-conditioning (HVAC) system [9,10]; meteorological conditions, e.g., wind speed and indoor/outdoor temperature differences [8,9,11]; and building characteristics, e.g., tightness and number of floors [8]. Opening windows can increase the ACR as much as 2 h^−1^, and an attic fan can increase ACRs by up to 1 h^−1^ [9]. Generally, effects due to temperature and wind are small [8,9] unless exterior doors and windows are closed [11]. Predictions of ACRs in residences show reasonable accuracy, e.g., the median absolute difference between predicted and measured ACRs in 31 detached homes was 40 to 50% [1].

Few studies have measured the spatial variation in pollutant concentrations within buildings using a sufficient number of samples and buildings to characterize variation. Incomplete mixing within rooms has been demonstrated in specialized applications, e.g., filtration for control of airborne pathogens [12]. In a single-story residence, one and two-compartment models yielded good agreement with measurements, and interzonal flows depended on whether a door separating adjacent rooms was open (allowing the house to be modeled as a single compartment) or closed (requiring two compartments) [13]. In occupied buildings, mixing and air flows will vary over time as doors and windows are opened and closed, ventilation systems are turned on and off, and as outdoor meteorological conditions change. As noted, low ACRs and/or little mixing can produce high pollutant concentrations if emission sources are present.

## 2. Objectives

The goals of this paper include: characterizing ACRs and interzonal airflows in a large sample of residences; identifying determinants of ACRs, including seasonal and meteorological influences; and characterizing interzonal transport, particularly between bedrooms and general living areas (excluding the studied bedroom). Additionally, the collected data along with mechanistic models are used to understand the impact on IAQ, in particular the sensitivity of ACRs and mixing assumptions on concentrations due to indoor pollutant sources. The information presented in this paper can aid exposure assessments and inform decisions regarding IAQ management strategies, including the use of filters, requirements for mechanical ventilation, and the effectiveness of source controls such as smoking bans.

## 3. Materials and Methods

### 3.1. Recruitment, Sampling Schedule, and Study Homes

Recruitment of study homes. A total of 126 households in Detroit, Michigan, USA (population 715,000) were recruited as part of a study examining the effectiveness of stand-alone room air filters and air conditioners in mitigating asthma symptoms in children. Study households were predominantly low income African American and Latino [14], and each had a child with asthma. Households were randomized to one of three groups: (1) a “control” group receiving only community health worker (CHW) home education visits regarding the child’s asthma (n = 37); (2) a “standard” intervention group receiving a stand-alone HEPA filter placed in the child’s bedroom and the CHW visits (n = 47); or (3) an “enhanced” intervention group receiving the filter, the CHW visits, plus an air conditioner (n = 42). Households entered the study on a rolling basis from March, 2009 to September, 2010, and each received a weeklong “baseline visit” and two or three follow-up or “seasonal” visits spaced three or four months apart. On most weeks, 6 to 10 homes were sampled. The present paper reports on a total of 346 weeklong household visits to the 126 homes.

The study was conducted using a community-based participatory research approach by the Community Action Against Asthma (CAAA) partnership. All procedures were approved by The University of Michigan Institutional Review Board. Further details on the study design and exposure assessment activities are provided elsewhere [15].

#### 3.1.1. Walkthrough and Caregiver Surveys

A walkthrough audit of each house was completed to collect information on its characteristics and conditions, including type of heating and cooling system, evidence of water damage, mold and number of windows, and information about the child’s bedroom, sleeping and playing areas. Participants also completed surveys during baseline and seasonal visits that included questions about health status, features of their home, and indoor PM-emitting activities (e.g., frequency of cigarette smoking, cooking, and vacuuming). For a few participants, some survey or walkthrough data are missing, e.g., due to moves or refusals.

#### 3.1.2. Home and Household Characteristics

Characteristics of the study homes have been summarized previously [15]. Volumes of the houses and bedrooms averaged 368 ± 143 m^3^ (median = 360 m^3^, n = 121) and 28 ± 12 m^3^ (median = 25 m^3^, n = 122), respectively; floor areas averaged 147 ± 58 m^2^ (median = 146 m^2^, n = 121), and the children’s bedroom averaged 11 ± 5 m^2 ^(median = 10 m^2^, n = 122). Most homes (88%) used forced air heating systems, 30% had central air conditioners, 27% had exhaust fans, and 47% used furnace filters. The average occupancy of the homes was 1.7 ± 0.8 adults and 2.4 ± 1.4 children. Over half (60%) of the households included adult smokers according to the caregiver survey, and environmental tobacco smoke was detected (by the use of tracers) in 23 homes. Nearly half (44%) of the caregivers reported using vacuum cleaners, and all of the child’s sleeping area had been cleaned in the past two weeks by vacuuming, sweeping or dusting.

### 3.2. ACR, Interzonal Flow, and PM Measurements

**Figure 1:**
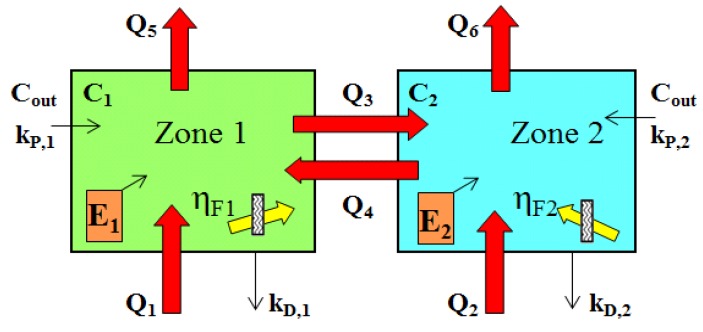
Configuration of the well mixed two-zone model, also, schematic of the two zone IAQ model, showing flows Q_1_ to Q_6_ (m^3^·h^−1^); concentrations C_out_, C_1_ and C_2 _(mg·m^−3^); emission rates E_1_ and E_2_ (mg·h^−1^); particle penetration factors k_P,1_ and k_P,2 _(dimensionless); deposition loss rates k_D,1_ and k_D,2 _(h^−1^); and capture efficiency of filters η_F1_ and η_F2_ (dimensionless).

Ventilation and air quality measurements were concurrently obtained in the living area and the child’s bedroom of each house during baseline and each seasonal assessments (summer was defined as June, July and August; fall as September, October and November; winter as December, January, February, and spring as March, April and May.) ACRs were estimated using the multizone constant injection method [16,17], which allows determination of “local” ACRs. The method used two different perfluorocarbon tracers (PFTs) and concentration measurements of the tracers in both zones. Two passive emitters of hexafluorobenzene (HFB) were placed in the living area, and two emitters of octafluorotoluene (OFT) in the sleeping area. Emitters were typically placed in opposite corners of rooms, releasing the PFT at a constant rate over the weeklong sampling period. PFT concentrations were measured using passive samplers at central locations in the two rooms, placed away from the emitters [18]. The passive samplers were analyzed using thermal desorption, cryofocusing and GC-MS analysis [19]. Figure 1 depicts flows Q_1_ to Q_4_ determined by this method; flows Q_5_ and Q_6_ are obtained by flow balance. Note that zone 1, called the living area in this paper, refers to all rooms other than the (study) bedroom.

ACRs for the house and bedroom, and flows between these zones, were determined using the measured concentrations and the volumes of the house and bedroom as follows [20]:

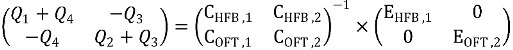
(1)
where Q_1_ and Q_2_ = air flows into zone 1 and 2, respectively, from outdoors (m^3^·h^−1^); Q_3 _= air flow rate from zone 1 to 2 (m^3^·h^−1^); Q_4 _= air flow rate from zone 2 to 1 (m^3^·h^−1^); C_HFB,1_ and C_OFT,1_ = concentrations of PFT tracers HFB and OFT in zone 1 (mg·m^−3^); C_HFB,2_ and C_OFT,2_ = concentrations of HFB and OFT in zone 2 (mg·m^−3^); and E_HFB,1_ and E_OFT,2_ = emission rates of HFB and OFT in zones 1 and 2 (mg·h^−1^), respectively. This result assumes that outdoor PFT concentrations are zero, and that the PFTs are inert (removed only by airflows and not by settling, deposition, filtration or reaction). The ACR_i_ (h^−1^) in zone *i* was calculated as Q_i_/V_i_ (i = 1,2), where V_i_ = volume of zone i (m^3^).

Interzonal flows. Interzonal flows transport pollutants between zones, e.g., cigarette smoke emitted in the living area that is brought to the bedroom. Interzonal flows Q_3_ and Q_4_ from the two zone model are expressed as interzonal flow proportions 𝛼_HB_ and 𝛼_BH_ (dimensionless, ratios between 0 and 1):

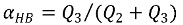
(2)

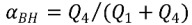
(3)
where 𝛼_HB_ = fraction of the air coming into the bedroom that arises from the (remainder of the) house, and similarly, 𝛼_BH_ = fraction of house air coming from the bedroom. These proportions provide a simple way to compare the magnitude of interzonal flows among buildings of different sizes.

Indoor and outdoor measurements. PM concentrations in bedrooms were measured as sequential 24-h filter samples during the sampling week collocated with the PFT samplers in each season. As detailed elsewhere [15], PM samples were collected at 15 L/min on 1 µm-rated PTFE filters installed in static-free polypropylene cassettes (Omega Specialty Instruments Co., Houston, TX, USA) for gravimetric analysis. The inlets on these cassettes are not designed to be size selective, and they essentially capture the total suspended particulate (TSP) fraction. In addition, particle number counts (PNCs) in 0.3–1.0 µm and 1–5 µm dia size ranges were measured continuously using an optical particle counter (GT-521, MetOne, Grants Pass, OR, USA). PM concentrations and 0.3–1.0 µm PNCs were significantly correlated [15]. The PM data were reduced to weeklong averages.

Outdoor PM_2.5_ measurements were obtained from air quality monitoring sites in Detroit selected to be representative of population exposure. These included daily data from four sites (Allen Park, Ambassador Bridge, Dearborn, Newberry School), and every third day data from five additional sites (Southwest High School, Linwood, East 7 Mile, Livonia, Wyandotte). These sites were operated by the Michigan Department of Natural Resources and the Environment using protocols that followed standard federal reference methods. Meteorological data, including wind speed, direction, temperature, humidity, and barometric pressure, were obtained from the Detroit City Airport site located near the middle of the study area.

In addition to the PFT tracers, the same passive samplers measured two tracers of environmental tobacco smoke (ETS), *i.e.*, 2,5-dimethylfuran and 3-ethenylpyridine used to confirm the presence of ETS, along with about 100 other volatile organic compounds (VOCs), e.g., naphthalene, BTEX (sum of benzene, toluene, ethylbenzene and xylenes), and total volatile organic compounds (TVOC, sum of target compounds) [15,18,20,21]. These samplers were placed in bedrooms and living areas for a 1-week period, and analyzed using thermal desorption, gas chromatography and mass spectrometry. Temperatures and relative humidity also were monitored in both bedrooms and living rooms, and CO_2_ was monitored in the bedroom using an infrared sensor. These variables were monitored continuously and reduced to 1-week averages.

Quality assurance (QA). PFT, VOC and ETS tracer measurements used duplicate samplers and showed good agreement, *i.e.*, replicate precision averaged 11 ± 12% for the PFTs, 15 ± 16% for VOCs, and 14 ± 13% for the ETS tracers. Field blanks for passive sampling tubes were deployed at each household each week, and showed negligible contamination. Emitters were weighed periodically to determine emission rates, and samplers were temperature corrected. ACR measurements that were excessively large (≥10 h^−1^) or unrealistically small (≤0.1 h^−1^) probably resulted from incomplete mixing or other reasons, and thus were omitted from analyses. (As shown later, such values constituted a very small fraction of measurements.) Further description of QA is provided elsewhere [15].

### 3.3. IAQ Modeling

Two zone IAQ model. PM concentrations from indoor and outdoor sources were predicted using a two zone model [4] that represents the bedroom and the remainder of house (Figure 1). Each zone can have an internal emission source that adds pollutants, and an (free-standing) air filter that removes pollutants. The mass balances in the two zones are similar:

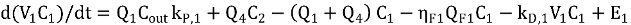
(4)

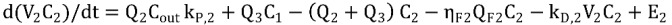
(5)
where C_1_ and C_2_ = PM concentrations in zones 1 and 2, respectively (μg·m^−3^); t = time (h); C_out_ = outdoor PM concentration (μg·m^−3^); η_F1_ and η_F2_ = PM capture efficiency of filters, if any, in zones 1 and 2 (dimensionless); Q_F1_ and Q_F2_ = filter air flow rate (m^3^·h^−1^); k_D,1_ and k_D,2_ = deposition loss rate for zones 1 and 2 (h^−1^); k_P,1_ and k_P,2_ = particle penetration factor for zones 1 and 2 (dimensionless); E_1_ and E_2_ = emission rates in zones 1 and 2 (mg·h^−1^), respectively; and flows Q_1_ to Q_4_ and volumes V_1_ and V_2_ were defined earlier. PM removal by a forced air system was not considered. Most of these parameters can vary by residence, time and season.

Given known flows and emission rates, and assuming steady-state conditions, Equations (4) and (5) can be solved for concentrations C_1_ and C_2_ as:

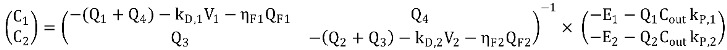
(6)


Scenarios and model parameters. Several scenarios were simulated in order to predict indoor PM concentrations and demonstrate the migration of PM between zones. (The same scenarios are used for the sensitivity analysis, described below). Scenario 1 considered an emission source (e.g., smoking) in zone 1, e.g., the living area. Scenario 2 moved the source to zone 2, e.g., the bedroom. Finally, scenario 3 considered only outdoor emission sources (no indoor sources). Cigarette smoking was the sole indoor emission source considered, and 10 cigarettes were assumed to be smoked daily, giving an average PM emission rate E_1_ of 7.5 mg h^−1^ based on an emission factor of 18 mg cigarette^−1^ [13,21]. This scenario is relevant for other indoor sources with equivalent PM emissions. No other emission source, other than PM infiltration from outside air, was considered. In residences, many other indoor sources other than ETS make significant contributions to PM concentrations, e.g., cooking, cleaning, grooming, dusting, vacuuming, and resuspension. These scenarios did not use filters.

The three scenarios had several variants. A free standing filter HEPA filter in the bedroom was added to the three scenarios (designated as scenarios 1F, 2F and 3F). Also, emission rates were adjusted to match PM concentrations measured in the field study, both with and without filters (scenarios 1M, 1FM, 2M and 2FM). In each case, PM concentrations in the bedroom and living area were predicted using Equation (6).

A set of nominal model parameters was selected to represent the study homes. These used the average volumes of the houses and bedrooms V_1_ and V_2_, and air flows Q_1_, Q_2_, Q_3_ and Q_4_ scaled to the average house volume (based on average ACRs and interzonal flow proportions, and using only those homes with valid Q_1_, Q_2_, Q_3_ and Q_4_ measurements). Filter airflow rate Q_F2_ was set to an effective average value, which depended on the filter fan speed and proportion of time used. We assumed the lowest speed (400 m^3^·h^−1^), weighted by the average filter usage among study participants (70%) [22], thus obtaining Q_F2_ = 280 m^3^·h^−1^ (assuming continuous use of the filter). For filter efficiency, η_F2_ was set to 1.0, typical of the high efficiency (HEPA) filters used [23]. No air filter was installed in zone 1, thus Q_F1_ was set to 0 m^3^·h^−1^. Outdoor PM concentration C_out_ was set to 11 μg·m^−3^, the average value among nine Detroit monitoring sites over the study period [22,24]. Deposition velocity k_D_ depends on PM properties (e.g., particle size and density), air turbulence, and room characteristics (e.g., volume to surface area ratio), and values from 0 to 3.6 h^−1^ have been proposed based on theoretical and experimental values [23,25,26,27]. We set k_D,1_ and k_D,2_ to 0.2 h^−1^. Particle penetration rates depend on particle size and environmental conditions, and a wide range, 0.001 to 1.0, has been suggested [28,29,30]. We assumed k_P,1_ and k_P,2_ were 0.5. Lastly, we matched observed results for C_2_ in four cases (with and without ETS detection, and with and without the filter) by adjusting emission rates E_1_ and E_2_.

### 3.4. Data Analysis

Sensitivity analysis. The influence of parameters in the two zone IAQ model is shown using sensitivity analyses for scenarios 1, 1F, 2, 2F, 3 and 3F using the nominal parameters just described (and assuming 10 cigarettes smoked daily indoors except in scenario 3). Results were expressed for each parameter i using the relative sensitivity RS_i_ (dimensionless):

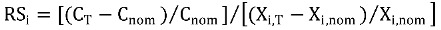
(7)
where C_T _= PM concentration obtained for a 10% increase in parameter X_i_ (*i.e.*, Q_1_, Q_2_, Q_3_, Q_4_, E_1_, E_2_, V_1_, V_2_, η_F2_ Q_F2_, k_D,1_, k_D,2_, k_P,1_C_out_ and k_P,2_ C_out_) from its nominal value for the scenario, X_i,nom_; and C_nom_ = PM concentration for nominal values of parameter X_i,nom_. Thus, RS_i_ represents the change in PM levels relative to a change in input X_i_, while holding other parameters at nominal values. 

Statistical analysis. Cumulative distributions of ACRs were plotted and tested for normality or lognormality using Anderson-Darling goodness-of-fit tests. For parameters fitting either distribution, the variability was apportioned to season/year and house effects using a variance components analysis, computed using the MIXED and NESTED procedures in SAS (v9.1.3, SAS Institute, Cary, NC, USA) [31]. Key parameters were tested by intervention group using Kruskal-Wallis nonparametric tests for differences in medians, and F and Tukey’s tests for means. Variables from the walk-though and caregiver surveys that might be plausibly associated with IAQ and air flow parameters were selected for analysis. Spearman correlation coefficients were used to examine associations between ACRs, house and occupant characteristics.

## 4. Results and Discussion

### 4.1. ACRs in Residences and Bedrooms

**Figure 2:**
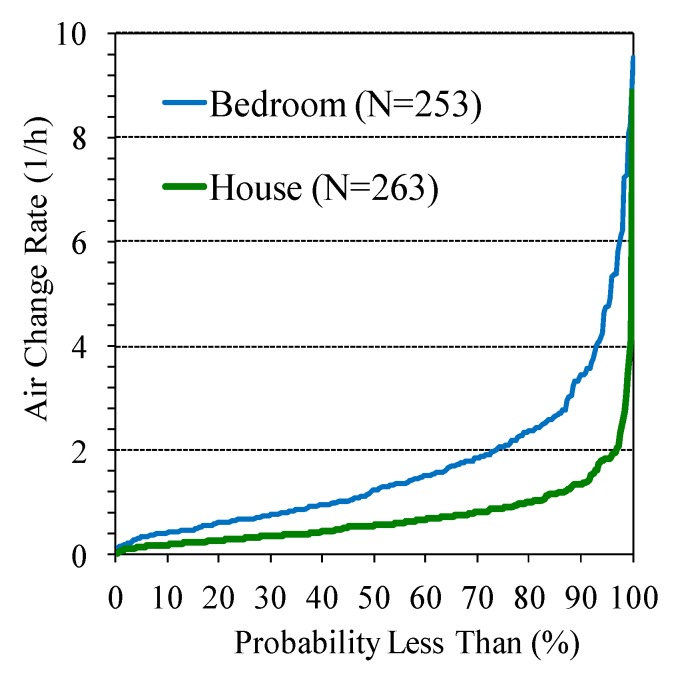
Cumulative distributions of ACRs in houses and bedrooms.

In houses, ACRs averaged 0.73 ± 0.76 h^−1^ (median = 0.57 h^−1^, n = 263) and the interquartile range (IQR) was 0.32 to 0.90 h^−1^. The distribution of ACRs (Figure 2) show that most values fell in a narrow range, e.g., only 10% of measurements were below 0.2 h^−1^ and 1% exceeded 4 h^−1^, and measurements were lognormally distributed (*p* = 0.32, Anderson-Darling test). The house ACRs exclude flows between two zones, *i.e.*, only outside air entering the zone is considered. ACRs in the control group (median = 0.56 h^−1^, n = 74), standard intervention group (0.60 h^−1^, n = 101) and enhanced intervention group (median = 0.54 h^−1^, n = 88) did not differ statistically (*p* = 0.75, Kruskal-Wallis test).

In bedrooms, ACRs averaged 1.7 ± 1.5 h^−1^ (median = 1.2 h^−1^, n = 253) and the IQR was 0.68 to 2.07 h^−1^. Overall, bedroom ACRs were approximately twice that seen for the house, suggesting that windows were frequently open (at least in the summer) or that the bedrooms were relatively “leaky”. Most of the sampled bedrooms were small (volume of only 28 ± 12 m^3^), and all had at least one (and sometimes several) operable windows and exterior walls. The large exterior wall-to-volume ratio in these bedrooms may increase air infiltration due the driving forces of the wind and stack effects (indoor-outdoor temperature differences) compared to the house as a whole, and thus yield a higher ACR. The distribution of bedroom ACRs, also shown in Figure 2, was neither normal nor lognormal, thus the variance proportions analysis was not conducted. These ACRs did not differ by group (*p* = 0.40; median levels in control, standard, and enhanced intervention groups were 1.31 h^−1^ [n = 69], 1.33 [n = 99], and 1.03 [n = 85], respectively). ACRs in bedrooms and houses were weakly, but significantly correlated (Spearman r = 0.21, *p* = 0.001).

Because ACRs did not differ by intervention group, these data were pooled for subsequent analyses. Standard and enhanced interventions were merged and are denoted as the “with filter” group; the control group is called “without filter” group. Four observations were excluded as outliers (3 bedroom ACRs ≥ 10 h^−1^ and 1 bedroom ACR ≤ 0.1 h^−1^), representing only 1.6% of the collected data.

**Table 1:** Air change rates in the house and child’s bedrooms by season.

Outcome	Season	Without filter				With filter				All groups			
		N	Average	SD	Median	N	Average	SD	Median	N	Average	SD	Median
ACR_H_ (h^−1^)	Spring	21	0.46	0.24	0.37	58	0.61	0.62	0.41	79	0.57	0.55	0.40
	Summer	33	0.91	1.53	0.46	49	0.69	0.45	0.60	82	0.78	1.03	0.58
	Fall	8	0.78	0.50	0.60	44	0.78	0.65	0.60	52	0.78	0.63	0.60
	Winter	12	0.97	0.61	0.79	38	0.84	0.64	0.72	50	0.88	0.63	0.74
	All	74	0.78	1.08	0.55	189	0.72	0.60	0.57	263	0.73	0.76	0.57
	*p*-value *	0.060				**0.037**				**0.002**			
ACR_B_ (h^−1^)	Spring	20	1.41	1.05	0.96	56	1.19	0.81	1.05	76	1.25	0.87	1.00
	Summer	29	1.88	1.84	1.34	46	2.27	2.16	1.74	75	2.12	2.03	1.50
	Fall	8	2.18	1.92	1.30	45	1.50	1.27	1.14	53	1.60	1.39	1.23
	Winter	12	1.67	1.02	1.43	37	1.65	1.29	1.30	49	1.65	1.22	1.30
	All	69	1.74	1.52	1.31	184	1.63	1.49	1.18	253	1.66	1.50	1.23
	*p*-value *	0.822				**0.048**				**0.049**			

* *p*-value from Kruskal-Wallis test for differences among homes by seasons.

ACRs differed by season (Table 1). House ACRs were lowest in spring and highest in winter, with an overall variation of about 53% based on median seasonal values. Based on the variance proportions analysis, temporal variation (year and season) explained 74% of the total variation, and house-to-house variation explained the remainder, 26%, showing a strong need for seasonal measurements. Homes with filters showed smaller and marginally significant seasonal changes. The reason for differences between the groups with and without filters is not clear. While not statistically significant, homes with filters were slightly smaller (volume = 362 ± 146 m^3^* versus* 371 ± 124 m^3^) and younger (average age was 54 *versus* 61 years, though definitive ages were known for only 39 of the homes) than homes without filters. Occupants in homes receiving filters were instructed to try to keep bedroom doors closed or partially closed (to maximize the filter’s effect), but compliance with this instruction was likely low.

In bedrooms, ACRs also varied by season, and again the lowest values occurred in spring. However, the highest ACRs in bedrooms occurred in summer (excluding the small number of homes tested without filters), probably reflecting the use of air conditioners, opened windows, or fans to cool the bedrooms. Thus, compared to ACRs in houses, ACRs in the bedrooms were higher and showed different seasonal trends. As discussed below, these are important and striking results that are not well recognized in the literature.

Comparison to the literature. The house ACRs measured in the present study are generally comparable to those reported in several recent studies, all of which used the constant injection tracer gas method. In the Detroit Exposure and Aerosol Research Study (DEARS), median ACRs in 120 households in Detroit ranged from 0.7 to 1.4 h^−1^, depending on season [32]. In another southeast Michigan study, but in the more suburban and affluent communities of Ann Arbor and Ypsilanti, ACRs were lower, averaging 0.43 ± 0.37 h^−1^ (n = 15) [33]. Homes in nearby Windsor, Ontario, Canada were also “tighter” than the Detroit homes, with a geometric mean ACR in winter of 0.32 h^−1^ (95th percentile confidence interval [CI]: 0.26 to 0.40 h^−1^; n = 32), and only 0.19 h^−1^ (CI: 0.15 to 0.24 h^−1^; n = 42) in summer [34]. In the Relationship among Indoor Outdoor and Personal Air (RIOPA) study, which measured approximately 100 residences each in Elizabeth, NJ, Houston, TX and Los Angeles, CA, the median ACR was 0.71 h^−1^ (n = 506), and ACRs differed by city (0.87, 0.88 and 0.47 h^−1^, respectively), as well as by season [35]. An analysis of the 1997 U.S. Department of Energy Residential Energy Consumption Survey, which included 140 single-family houses in 19 cities, showed a median ACR of 0.44 h^−1^ (range from 0.26 to 0.58 h^−1^) [36]. Overall, the ACRs in the present study are somewhat higher than those found in similar (northern) climates, reflecting the age, lack of weatherization, and often poor condition of the Detroit housing stock, factors associated with increased building leakage area. However, ACRs in the study homes are lower than for residences located in more moderate climates, which can use natural ventilation more extensively.

ACRs in bedrooms. There are few reports of ACRs measured in bedrooms. In Europe, low ACRs have been found in bedrooms, for example, the geometric mean ACR in children’s bedrooms in Odense, Denmark was 0.46 ± 2.09 h^−1^ (n = 500) [37,38]. The mean ACR in children’s bedrooms of single family homes in Sweden ranged from 0.31 to 0.47 h^−1^ (n = 390), and depended on the ventilation system, construction period, foundation type and number of floors [39]. Notably, northern European residences typically have very low ACRs, e.g., ACRs in the single-family houses in the Swedish study just noted averaged 0.36 h^−1^ (n = 333) [39]. Due to the substantial differences in building air tightness, ventilation, climate and other factors, ACRs in the US and Europe were not expected to be similar. Nonetheless, it is interesting to note that bedrooms in the Detroit homes have much higher ACRs than the remainder of the house and thus likely to be rather drafty in winter. In contrast, the two European studies show similar ACRs in houses and bedrooms.

A number of studies reporting ACRs, especially those reporting results for bedrooms, have used a single PFT or SF_6_ tracer in all zones or rooms [9], or have used occupant-generated CO_2_ as a tracer [40]. These studies can have significant limitations and potential biases that are eliminated in the present study by the use of multiple PFT tracers.

Temporal variability. Seasonal differences in ACRs have been shown in many studies. As noted earlier, ACRs in homes in Windsor, Canada increased significantly in winter. In a nonsmoking household in Boston, MA, ACRs measured for one or two 6-day periods in two seasons using SF_6_ increased greatly in summer (mean = 3.80 h^−1^) when windows and doors were opened compared to winter (0.15 h^−1^); two other houses monitored in this study did not show seasonal differences [41]. In the RIOPA study, ACRs in Houston homes decreased in the summer cooling season (median = 0.37 h^−1^) compared to winter (0.63 h^−1^); Los Angeles showed the opposite trend where ACRs increased in summer (1.13 h^−1^) compared to winter (0.61 h^−1^); and homes in Elizabeth showed similar ACRs in heating (median = 0.88 h^−1^) and cooling (0.63 h^−1^) seasons [35]. An older (1982–1987) study of 2,844 homes across the USA showed significant differences in ACRs by region, but considering all homes, ACRs were lowest in fall (average = 0.41 ± 0.58 h^−1^), highest in summer (1.50 ± 1.53 h^−1^), and comparable in spring (0.65 ± 0.57 h^−1^) and winter (0.55 ± 0.46 h^−1^) [42].

Seasonal variation in ACRs is driven by indoor-outdoor temperature gradients, wind speeds, occupant behavior, and building characteristics including the presence of air conditioners. ACRs typically increase in winter in regions with heating needs (like Michigan and Canada) due to greater indoor/outdoor temperature differences and higher wind speeds, and the stack effect (due to temperature differences) is enhanced in multistory buildings [1,11,34]. In summer and in warm climates, air conditioner use can lower ACRs, as seen in Houston, while in moderate climates or when temperatures are mild, natural (and sometimes mechanical) ventilation promoted by opened windows and doors increases ACRs [9,35,41]. In the present study, winters are typically cold and moderately windy, which increases ACRs; summers can be hot and humid, and air conditioner use is common. However, summers in the study period were cooler than normal and only 30% of the Detroit homes had central air conditioning, thus air conditioner use was less frequent and probably not representative of the broader region. For the house, ACRs were highest in winter and lowest in the spring when indoor-outdoor temperature rates were small. As noted earlier, bedrooms showed a different trend: again ACRs were lowest in spring, but ACRs were highest in summer, likely reflecting natural or enhanced (fans and air conditioners) ventilation.

Factors associated with ACRs. The house and occupant characteristics associated with ACRs are shown in Table 2. House ACRs were negatively and significantly associated with house size (area and number of bedrooms), the presence of a central air conditioner, indoor CO_2_ and VOC concentrations, and the presence of cigarette smokers. ACRs were positively associated with recent sweeping and dusting, and indoor PM concentrations. Similar results were found for bedroom ACRs with the addition of negative correlations with outdoor wind speed. Daily average wind speeds during the sampling period ranged from 1.8 to 5.1 m/s, and wind speed was negatively but not statistically significantly correlated to the house ACRs. The lack of significant associations with the meteorological variables is likely a result of the weeklong ACR measurements, which average over shorter-term fluctuations in these variables, as well as wind and sun sheltering, which diminishes the agreement with airport meteorological data.

**Table 2:** Association between ACRs and house and building/occupant characteristics. Spearman correlation coefficients used for continuous variables, and Kruskal-Wallis tests for categorical variables. Statistically significant associations (*p* < 0.05) in bold. PNC = particle number count; CSA = child’s sleep area (e.g., bedroom); * C = continuous variable; I = indicator variable; M = multilevel categorical variable.

Variable			Type *	ACR_H_			ACR_B_		
				N	Correlation Coefficient	*p*-value	N	Correlation Coefficient	*p*-value
House	Floor area		C	256	**−0.363**	**<0.001**	247	−0.073	0.255
	Forced air heating system/others		I	225/29	-	0.943	217/29	-	0.522
	No. of bedrooms		C	254	**−0.135**	**0.031**	246	−0.040	0.533
	Present/not present central AC		I	86/168	-	**0.012**	83/163	-	0.620
	Present/not present ventilation fan		I	75/188	-	0.374	73/180	-	0.789
	Furnace filter change frequency		C	117	0.046	0.620	114	0.111	0.238
Child’s sleeping area	Floor area		C	257	−0.097	0.119	248	−0.083	0.192
	Room volume		C	257	−0.069	0.273	248	−0.088	0.167
	Number of windows		C	248	−0.016	0.805	241	0.015	0.815
Occupancy	No. of adults		C	263	−0.076	0.222	253	−0.070	0.268
	No. of children		C	263	0.079	0.202	253	−0.021	0.741
	Dogs present		C	83	0.199	0.072	81	−0.005	0.961
	Cats present		C	29	−0.020	0.917	26	−0.158	0.440
	Present/not present either dogs or cats		I	102/161	-	0.651	99/154	-	0.638
Smoking	Never any smokers indoors/smokers indoor		I	148/115	-	**0.029**	142/111	-	0.842
	Any smokers in household/no smokers		I	153/110	-	**0.023**	148/105	-	0.174
	Number of smokers		C	263	**0.162**	**0.008**	253	0.079	0.212
Cleaning	Use/not use a vacuum cleaner		I	117/22	-	0.416	115/21	-	0.246
	Vacuumed CSA in the last 2 weeks		C	117	−0.004	0.967	115	0.014	0.879
	Swept/dusted CSA in the last 2 weeks		C	263	**0.212**	**0.001**	253	0.164	**0.009**
Air pollutants	Outdoor	PM_2.5_ (µg/m^3^)	C	210	0.092	0.184	201	0.132	0.061
	Indoor	PM (µg/m^3^)	C	225	**0.152**	**0.023**	216	**0.146**	**0.032**
		0.3−1.0 µm PNC (#/liter)	C	224	0.077	0.248	214	**0.216**	**0.001**
		1-5 µm PNC (#/liter)	C	224	0.055	0.415	214	0.064	0.352
		CO_2_ (ppm)	C	241	**−0.246**	**<0.001**	233	−0.334	<0.001
		Naphthalene (µg/m^3^)	C	252	**−0.188**	**0.003**	242	−0.095	0.142
		BTEX (µg/m^3^)	C	252	**−0.345**	**<0.001**	242	**−0.199**	**0.002**
		TVOC (µg/m^3^)	C	252	**−0.311**	**<0.001**	242	**−0.184**	**0.004**
Meteorology	Average temperature		C	263	−0.062	0.318	253	0.029	0.647
	Minimum relative humidity		C	263	**0.201**	**0.001**	253	0.069	0.274
	Average daily station pressure		C	263	0.079	0.199	253	**0.188**	**0.003**
	Resultant wind direction		C	263	0.017	0.786	253	−0.024	0.701
	Average wind speed		C	263	−0.090	0.147	253	**−0.132**	**0.035**
Season (Spring, Summer, Fall, Winter)			M	-	-	**0.002**	-	-	**0.049**

The associations between ACRs and the various factors are generally consistent with expectations. For example, CO_2_ can be an indicator of occupancy and ventilation, and CO_2_ levels generally increase with low ACRs. Similarly, concentrations of VOCs and other pollutants with indoor sources will increase with low ACRs. Individuals may open windows and doors when sweeping and dusting, which will increase ACRs; these activities are also likely to entrain dust and thus increase PM concentrations. The Detroit data does not include variables reflecting opened windows and doors, a strong determinant of ACRs as shown in studies in Ohio [11], California and Virginia [8], Redwood City and Watsonville, CA [43], Columbus, OH [11], Odense, Denmark [37], and Boston, MA [41]. As noted earlier, wind speed and indoor-outdoor temperature differentials are important determinants of ACRs [1,8,9,11]. ACRs decrease in homes with central air conditioning, especially in summer, reflecting closed windows [22]. Other factors affecting ACRs, which have been incorporated in several predictive models, include the presence of an attached garage [34], ﬂoor area, envelope airtightness (often related to the house age), number of ﬂoors, foundation type, presence of a forced air distribution system, the number of units in apartment buildings, climate and city/region [36].

### 4.2. Interzonal Flows

A seasonal analysis of interzonal flows between the house and bedroom, including proportions 𝛼_HB_ and 𝛼_BH_, is shown in Table 3. Overall, 55 ± 18% of the air entering the bedroom came from the house; the balance directly entered the bedroom from outdoors. For the house’s main living area, an average of 26 ± 20% of air entered from the child’s bedroom; most air entered directly from outdoors. While some change in these proportions was seen by season, intervention group and the presence of central air conditioning and smokers, these differences were not statistically significant.

**Table 3:** Proportion of interzonal flows in the house and bedroom by season and intervention group.

Outcome	Season	Without filter				With filter				All groups			
		N	Average	SD	Median	N	Average	SD	Median	N	Average	SD	Median
𝛼_HB_	Spring	20	0.55	0.18	0.60	55	0.53	0.16	0.53	75	0.54	0.17	0.54
	Summer	23	0.57	0.21	0.58	38	0.51	0.19	0.53	61	0.53	0.20	0.53
	Fall	7	0.46	0.25	0.52	42	0.60	0.18	0.62	49	0.58	0.20	0.61
	Winter	11	0.55	0.21	0.58	38	0.54	0.18	0.53	49	0.54	0.18	0.55
	All	61	0.55	0.20	0.58	173	0.55	0.18	0.54	234	0.55	0.18	0.55
	*p*-value *	0.804				0.189				0.525			
𝛼_BH_	Spring	20	0.30	0.19	0.30	55	0.23	0.17	0.20	75	0.25	0.17	0.22
	Summer	23	0.38	0.28	0.31	38	0.27	0.24	0.21	61	0.31	0.26	0.26
	Fall	7	0.24	0.26	0.24	42	0.24	0.17	0.20	49	0.24	0.18	0.20
	Winter	11	0.21	0.17	0.16	38	0.25	0.15	0.24	49	0.24	0.15	0.22
	All	61	0.31	0.23	0.27	173	0.25	0.18	0.20	234	0.26	0.20	0.22
	*p*-value *	0.249				0.942				0.753			

* *p*-value from Kruskal-Wallis test for differences among homes by seasons.

Bedroom ACRs were negatively correlated with 𝛼_HB _(r = −0.36, *p* < 0.001) and 𝛼_BH _(r = −0.30, *p* < 0.001), indicating that tighter homes (low house and bedroom ACRs) had relatively higher flows between zones. In homes with forced air central air conditioning and heating systems, for example, windows are normally closed and air circulates rapidly throughout the house. Conversely, homes with higher ACRs tend to have flows that are decoupled between zones. While our study does not indicate the causes, this pattern could be explained by local exchange that occurs, for example, as windows are opened in several rooms and on several floors, causing separate (decoupled) air flow patterns in different portions of the building.

As noted, few measurements of airflows within residences or other buildings are available [4,5,13,44]. Short-term tests conducted in three homes with the ventilation system off showed that interzonal air flows (measured using a tracer gas) varied strongly as a function of door position or opening width [4,44]. Several modeling exercises have been completed. A two zone model for a single-story house in California, which gave reasonable fit between predicted and observed CO concentrations, showed interzonal air flow ratios of 0.976 (𝛼_BA_, proportion of room A’s intake air coming from room B) and 0.614 (𝛼_AB_, proportion of room B’s intake air from room A) after smoking a cigar for 15 min in the kitchen (emission rate of 60 mg/min) [13]. This house is not representative of Detroit homes, e.g., ACRs were very high (4.0 and 4.6 h^−1^ for the kitchen and living room, respectively), and the two zones had similar volumes (34 and 36 m^3^). An aerosol dynamics model using experimental data in a two zone test facility showed interzonal flows from 0.6 to 154 m^3^·h^−1^ from a non-smoking room (31 m^3^) to a smoking room (36 m^3^), and reverse flows from 1.1 to 163 m^3^·h^−1^, depending on the sealing between rooms, ventilation and filtration system [4]. Again, room volumes and configurations are not comparable to our study. Thus, the present study is believed to be one of the first reports of interzonal airflows within North American residences. (We previously reported on flows between attached garages and residences, along with several others) [33].

### 4.3. Scenario Analyses

Model parameters. Table 4 shows statistics of the measured parameters used by the two-zone model. As noted, interzonal flows Q_3_ and Q_4_ were positively correlated with outdoor airflows (Q_1_, Q_2_) and house volume, and interzonal flow Q_4_ was negatively correlated with bedroom volume and positively correlated with outdoor pollutant concentrations (data not shown).

**Table 4:** Statistics of airflows for house (Q_1_–Q_4_) and filter (Q_F2_), house and bedroom volumes (V_1_, V_2_), and outdoor PM concentrations (C_out_), including number of observations (N), average, standard deviation (SD), median, 25th, 75th and 90th percentile values.

Parameter	Unit	N	Average	SD	25th	Median	75th	90th
Q_1_	m^3^·h^−1^	234	242	272	109	179	287	446
Q_2_	m^3^·h^−1^	234	43	39	17	31	52	87
Q_3_	m^3^·h^−1^	234	57	76	23	41	71	102
Q_4_	m^3^·h^−1^	234	84	138	27	54	96	166
Q_F2_	m^3^·h^−1^	156	456	223	282	499	661	722
V_1_	m^3^	234	360	137	261	359	434	495
V_2_	m^3^	234	28	11	22	25	29	36
C_out_	μg·m^−3^	179	11	4	8	10	14	17

The nominal parameters derived for modeling purposes, intended to be representative of the field data, were as follows. House and bedroom volumes V_1_ and V_2_ were set to study averages, 360 and 28 m^3^, respectively. Airflows Q_1_, Q_2_, Q_3_ and Q_4_, scaled to the mean house volume, were 263, 50, 60 and 94 m^3^·h^−1^, respectively. As described earlier, Q_F2_ was set to 280 m^3^·h^−1^, and C_out_ to 11 μg·m^−3^, k_D,1_ and k_ D,2_ to 0.2 h^−1^, and k_P,1_ and k_P,2_ to 0.5.

Scenario results. Table 5 shows PM concentrations predicted for all scenarios, along with average concentrations measured in bedrooms, both with and without filters, and with and without the detection of ETS. (As mentioned, PM measurements in the living area were unavailable.) Observed concentrations were considerably elevated if smokers were present, and greatly lowered with the filter [15,22].

In scenario 1 (emission sources in the living area), the nominal emission rate (10 cigarettes day^−1^) yielded PM concentrations of 24 and 15 µg·m^−3^ in the living area and bedroom, respectively; concentrations in the bedroom dropped greatly, to only 4 µg·m^−3^, when the HEPA filter was used (scenario 1F). This nominal case closely matched the field study results in predicting a 73% reduction of PM levels in the bedroom due to the filter, as compared to the 69 to 80% measured [15]. However, predicted PM concentrations were low compared to measurements. Matching observed concentrations (39 µg·m^−3^) when ETS was present (by adjusting the number of cigarettes) required 33.5 cigarettes day^−1^ if the filter was not present (scenario 1MT), and 85 cigarettes day^−1^ if the filter was present (25 µg·m^−3^, scenario 1FMT). Especially the latter cigarette consumption rate seems unreasonably high, and these two scenarios produced correspondingly high PM predictions in the living area (71 and 172 µg·m^−3^). Most likely, some smoking, cooking and other PM emissions occur in or near the bedroom (e.g., dust from bedding, resuspension and exfoliated skin) [45], which were not modeled in this scenario (emissions were assumed to occur in only the living area). When ETS was not present, PM emission rates equivalent to 21.5 and 37.5 cigarette day^−1^ matched concentrations measured in the bedroom without and with the filter, respectively (27 and 12 µg·m^−3^, scenarios 1M and 1FM). Like the results just discussed, these scenarios do not consider other emission sources of PM, which are certainly present in residences. Again, we note that the number of cigarettes smoked daily is used as an indicator of PM emissions. The actual PM emissions may be more benign than ETS, which has a unique nature and the potential for serious health impacts. 

**Table 5:** Average concentrations observed in homes and results of scenario analyses, including adjustment of emission rates to match observed concentrations. C_1_ and C_2_ are concentrations in living room (LA) and bedrooms (BR), respectively.

Case or Scenario		Condition	Emission Rate		No Filter		With Filter (F)	
Type	No.		(cig/day)	(mg·h^−1^)	C_1_ (LA) (µg·m^−3^)	C_2_ (BR) (µg·m^−3^)	C_1_ (LA) (µg·m^−3^)	C_2_ (BR) (µg·m^−3^)
Observed in Field Study	-	Houses with ETS	-	-	-	39	-	25
	-	Houses without ETS	-	-	-	27	-	12
Source in Living Area	1, 1F	Nominal rate	10.0	7.5	24	15	22	4
	1MT	Match to ETS without filter	33.5	25.1	71	**39**	64	10
	1FMT	Match to ETS with filter	85.0	63.8	172	92	157	**25**
	1M	Match to non-ETS without filter	21.5	16.1	47	**27**	43	7
	1FM	Match to non-ETS with filter	37.5	28.1	78	43	72	**12**
Source in Bedroom	2, 2F	Nominal rate	10.0	7.5	20	78	8	21
	2MT	Match to ETS without filter	4.7	3.5	12	**39**	6	10
	2FMT	Match to ETS with filter	12.0	9.0	24	93	9	**25**
	2M	Match to non-ETS without filter	3.0	2.3	9	**27**	5	7
	2FM	Match to non-ETS with filter	5.5	-	13	45	6	**12**
No Indoor Sources	3, 3F	Without filter	-	-	4	5	4	1

Scenario 2, where indoor emissions occur in only the bedroom, yielded considerably different results. In this case, the nominal emission rate (10 cigarettes day^−1^) without a filter produced 78 and 20 µg·m^−3^ in the bedroom and living area, respectively, and 21 and 8 µg·m^−3^ with the filter (scenarios 2, 2F). The bedroom concentration become quite elevated due to this room’s small volume (despite its relatively rapid ACR), and the relatively small fraction of air from the bedroom to the rest of the house limited concentrations in other living areas. Again, the HEPA filter provides good control of PM in the bedroom, reducing concentrations by 74%, and also in the rest of the house (which was not seen in scenario 1). Matching observed concentrations with ETS detection required the equivalent of 4.7 cigarettes day^−1^ without the filter, and 12 cigarettes day^−1^ with the filter (scenarios 2MT and 2FMT, respectively). When ETS was not detected, emission rates equivalent to 3 and 5.5 cigarette day^−1^ matched concentrations observed without and with the filter (scenarios 2M and 2FM, respectively). Compared to smoking in the living area (scenario 1), much lower emission rates in the bedroom are needed to match observed concentrations. 

Without indoor emission sources, scenario 3 shows that infiltration of outdoor PM gives living area and bedroom concentrations that are similar and low, 4 and 5 µg·m^−3^, respectively, without the filter. The bedroom concentration is minimal (1 µg·m^−3^) with the filter (scenario 3F). The PM_2.5_ infiltration factors (calculated as the ratio of the indoor to outdoor concentrations) were 0.36 and 0.45 in the living area and bedroom, respectively. The PM_2.5_ infiltration factor measured in other Detroit homes in DEARS was 0.70 ± 0.33, which included homes with and without indoor sources [32]. Since indoor sources can increase measured infiltration factors, and given the limitations of the two zone IAQ model and the use of several literature values in this study (*i.e.*, models were not fitted), the modeled infiltration factors are reasonable.

The actual emission rates, air flows and other factors affecting PM levels in the study homes almost certainly differed from the modeled scenarios, thus explaining some of the results and the apparent discrepancies in Table 5. In particular, specific emission rates and source locations are unknown, emissions likely occur in both zones simultaneously, most model parameters will vary in time, literature values of some parameters may not apply, and the considerable variation in house configurations is simulated only approximately using point estimates such as the mean. Further, several differences in house parameters were noted where ETS was detected, e.g., volumes of these houses and bedrooms (V_1_ = 329 ± 132 m^3^; V_2 _= 24 ± 5 m^3^; n = 49) were significantly smaller than those without ETS detection (V_1_ = 368 ± 138 m^3^, V_2_ = 29 ± 12 m^3^; n = 185, *p* = 0.026; *p* = 0.014, Mann-Whitney test). (No other differences were noted.) Additionally, occupants may have changed their behavior when monitored or if the HEPA filter was present.

Notably, most of the modeled scenarios show strong differences of PM concentrations in the two zones in the house. For strong emission sources in (only) the living area, concentrations in the bedroom are 43 to 47% lower than those in the living area without a filter, and 83 to 84% lower with a filter (scenarios 1 and 1F, respectively). For strong emission sources in (only) the bedroom, concentrations in the bedroom are 65 to 74% higher without the filter, and 31 to 65% higher with the filter (scenarios 2 and 2F). These differences are due to the limited interzonal flow rates and the strong emission source that boosts concentrations well above that due to the penetration of (contaminated) outdoor air. In all scenarios, the HEPA filter substantially lowers concentrations in the bedroom. Moreover, the filter substantially lowers living area concentrations if emissions occur primarily in the bedroom (scenario 2F). Without indoor sources, levels in the bedroom slightly exceed those in the living area, a result of relatively greater penetration and exchange rates in the bedroom. Thus, the high ACRs in bedrooms can be important for exposure estimation purposes, especially given the substantial fraction of time people spend in bedrooms. Of course, results depend on the choice of model parameters, which is analyzed next.

### 4.4. Sensitivity Analyses

The relative sensitivity (RS) of each model parameter for six scenarios is shown in Table 6. As expected, high sensitivity (RS = 0.54 to 0.94) is shown for indoor emission rates E_1_ and E_2_. This means that increasing E_1_ by 10%, for example, will increase concentrations in the living area and bedroom by 8.2 and 6.9%, respectively. In this case, results do not depend whether or not a filter is present. PM levels in the bedroom (C_2_) were also sensitive to air filter use (RS = −0.68), as noted earlier.

**Table 6:** Relative sensitivity of model parameters on predicted concentrations in living area (LR) and bedroom (BR) for six scenarios (scenario number in parentheses). Absolute values greater than 0.40 are shown in bold.

Para-meter	Units	Emissions in Living Area				Emissions in Bedroom				No Indoor Emissions			
		No Filter (1)		W/Filter (1F)		No Filter (2)		W/Filter (2F)		No Filter (3)		W/Filter (3F)	
		LR	BR	LR	BR	LR	BR		BR	LR	BR	LR	BR
Predicted concentrations													
C_1_	µg·m^−3^	24	-	22	-	20	-	8	-	4	-	4	-
C_2_	µg·m^−3^	-	15	-	4	-	78	-	21	-	5	-	1
Relative sensitivity													
E_1_	µg·h^−1^	**0.82**	**0.69**	**0.83**	**0.69**	-	-	-	-	-	-	-	-
E_2_	µg·h^−1^	-	-	-	-	**0.78**	**0.94**	**0.54**	**0.94**	-	-	-	-
Q_1_	m^3^·h^−1^	**−0.50**	**−0.42**	**−0.45**	−0.37	**−0.47**	−0.06	−0.18	−0.01	0.16	0.08	0.30	0.13
Q_2_	m^3^·h^−1^	−0.04	−0.29	0.00	0.05	−0.36	**−0.43**	−0.05	−0.09	0.02	0.08	0.03	**0.44**
Q_3_	m^3^·h^−1^	0.05	0.35	0.03	**0.69**	−0.35	**−0.41**	−0.06	−0.10	−0.01	−0.03	0.02	0.30
Q_4_	m^3^·h^−1^	−0.09	−0.08	−0.18	−0.15	**0.69**	0.09	0.36	0.02	0.01	0.01	−0.15	−0.06
C_out_k_P,1_	µg·m^−3^	0.16	0.13	0.16	0.13	0.19	0.03	**0.44**	0.03	**0.87**	**0.42**	**0.96**	**0.42**
C_out_k_P,2_	µg·m^−3^	0.02	0.18	0.01	0.18	0.03	0.03	0.02	0.03	0.13	**0.58**	0.04	**0.58**
k_D,1_	h^−1^	−0.19	−0.16	−0.17	−0.14	−0.19	−0.03	−0.17	−0.01	−0.19	−0.09	−0.17	−0.08
k_D,2_	h^−1^	−0.01	−0.05	0.00	−0.01	−0.05	−0.05	−0.01	−0.01	−0.01	−0.05	0.00	−0.01
V_1_	m^3^	−0.19	−0.16	−0.17	−0.14	−0.19	−0.03	−0.17	−0.01	−0.19	−0.09	−0.17	−0.08
V_2_	m^3^	−0.01	−0.05	0.00	−0.01	−0.05	−0.05	−0.01	−0.01	−0.01	−0.06	0.00	−0.02
η_F2_Q_F2_	m^3^·h^−1^	-	-	−0.03	**−0.68**	-	-	−0.39	**−0.68**	-	-	−0.05	**−0.68**

In Scenario 1, PM concentrations in the bedroom were sensitive to interzonal flow Q_3_ (living area to bedroom), especially if a filter was present (RS = 0.69 and 0.35, with and without filter, respectively). PM concentrations were relatively unaffected by deposition rate (k_D,2_) and bedroom volume (V_2_). In Scenario 3 where outdoor pollutants were the only PM source, outdoor airflows Q_1_ and Q_2_ were key variables, especially in the bedroom when filters were used, and particle penetration factor k_P,1_ and outdoor PM concentration C_out_ became among the most sensitive parameters (especially in the living area). The influence of k_P,2_ and C_out_ was considerably lower, a result of the much larger volume of the living area compared to the bedroom.

As noted, the importance of interzonal flows Q_3_ and Q_4_ depended on the scenario. Low interzonal flows will impede transfers between zones, however, exposure to ETS will likely occur throughout a house even if smoking is restricted to one room [46]. While some degree of isolation and lower ETS exposure can be attained by closing doors and opening windows in the room containing smokers [13], the typical house design and HVAC configuration in the USA can quickly deliver pollutants throughout a house [2]. Thus, isolation is incomplete and this strategy has limited effectiveness. The two zone models allow easy evaluation of such strategies.

The key outcome of the sensitivity analysis is the identification of those model parameters that most strongly affect results. Results of the two zone model show that among the many parameters, emission rates, air flows and air filter use significantly affect pollutant levels, and thus it is important to obtain the most accurate data for these parameters to accurately model concentrations and exposures in homes. The results also demonstrate that with strong and localized sources like cigarettes, or with the use of a free-standing air filter, the assumption that the house is a single zone with uniform concentrations will not yield accurate results for either the smoker in the main living area or an individual in a bedroom.

## 5. Strengths and Limitations

This is believed to be one of the first reports of air flows between bedrooms and general living areas in North American homes, and one of the few reports quantifying ACRs in bedrooms (the few European studies that have measured these parameters have only limited applicability to US and Canadian homes.) ACRs and interzonal flows are critical parameters for understanding the effect of localized pollutant sources and pollutant control strategies, such as the degree of isolation that might be accomplished by restricting smoking to a particular area. For exposure purposes, bedrooms are important given the amount of time individuals spend sleeping at home. Additionally, the reported ACRs and air flow data were collected from a large sample of occupied houses and reflect seasonal factors, and the influence of house and other characteristics were identified. The sensitivity analysis modeled a variety of emission scenarios, each with and without a PM filter. This information describes the magnitude and spatial variation of pollutant concentrations, important information pertaining to exposure estimates and risk assessments for vulnerable populations such as children with asthma exposed to ETS. In fact, our concern regarding such exposures was the basis for selecting the study homes, and the results show that a substantial fraction of children with asthma are exposed to ETS at home.

The study has limitations with respect to information on PM emissions and occupant activities that might affect ACRs and PM levels, e.g., amount and location of smoking, filter use patterns, and the opening and positioning of windows and doors (which can strongly affect ACRs and interzonal airflows). PM concentrations in the living area of homes were not measured, and the time- and space-averaged value used in the modeling does not reflect the variation expected. Literature values of PM penetration rates also may not apply. The measurements and models are based on steady-state assumptions, and short term fluctuations will be missed. The study sample consisted of primarily single family homes that were smaller and older than US averages, and some results are unlikely to represent conditions for other building types and climatic regimes. Additionally, the Detroit population is primarily low-income and African American, and some home characteristics and occupant behaviors may differ from those in more affluent areas elsewhere in the USA or region. Additionally, the reported parameters and model results may not reflect actual conditions if the governing parameters dramatically change over the day or week. The modeling considered only two zones (more may be needed) and, as noted earlier, neither considered PM removal by forced air systems nor the many PM sources found indoors other than ETS (or an equivalent source) isolated to a single zone. PM measurements outside of individual homes were not available, and the use of a city-wide ambient PM_2.5_ concentration may not reflect local conditions at individual homes. However, ambient levels were low relative to indoor levels and thus unlikely to affect results greatly. The use of averaged parameters in the sensitivity analysis also has limitations, e.g., the results may not reflect conditions that diverge greatly from the nominal case used. While the tracer gas measurements met QA targets, estimation of ACRs and interzonal flows involves a number of assumptions and uncertainties. A preliminary analysis using Monte Carlo analysis indicates that precisions of the flow estimates should be within 15 to 20%; further analyses are underway and will be reported on subsequently. Finally, the sensitivity analysis does not incorporate the correlation among the parameters, although this is not expected to alter results greatly.

## 6. Conclusions

This paper presents results and analyses of a large survey of homes in Detroit, Michigan investigating air exchange rates (ACRs) and interzonal flows, two important factors relevant to indoor air quality (IAQ), as well as energy and comfort. The studied households were primarily low income and minority, and each had a child with asthma. In the houses’ living area, air change rates (ACRs) averaged 0.73 ± 0.76 h^−1^ (median = 0.57 h^−1^, n = 263). In the child’s bedroom, ACRs were substantially higher, averaging 1.66 ± 1.50 h^−1^ (median = 1.23 h^−1^, n = 253). Seasonal trends of house and bedroom ACRs differed. ACRs were positively associated with recent sweeping and dusting, and indoor PM concentrations, and negatively associated with house size, the presence of a central air conditioner and smokers, indoor CO_2_ and VOC concentrations. Interzonal air flows were measured, and their proportion of the total flow tended to increase as ACRs decreased. Scenario and sensitivity analyses using a two zone simulation model identified the key factors affecting pollution levels, which included the emission source strength, location, presence of an operating air filter, ACRs and interzonal air flows; secondary factors were PM penetration factors, deposition rates and house and room volumes. Single zone models applied only for homes without localized indoor emission sources or that did not use room air filters; otherwise, we conclude that room-to-room differences in concentrations of pollutants like PM can be significant. The results in this paper can inform the development of strategies designed to improve air quality, and can be used to estimate concentrations and exposures needed for epidemiology and risk assessment purposes. 
